# Supplementary material for: Consequences of SUR2[A478V] Mutation in Skeletal Muscle of Murine Model of Cantu Syndrome
Source: Cells. 2021 Jul 15;10(7):1791. doi: 10.3390/cells10071791 (PMC8307364; doi:10.3390/cells10071791)
Supplement: Supplementary file 1 [file cells-10-01791-s001.zip › cells-1250024-supplementary.pdf]

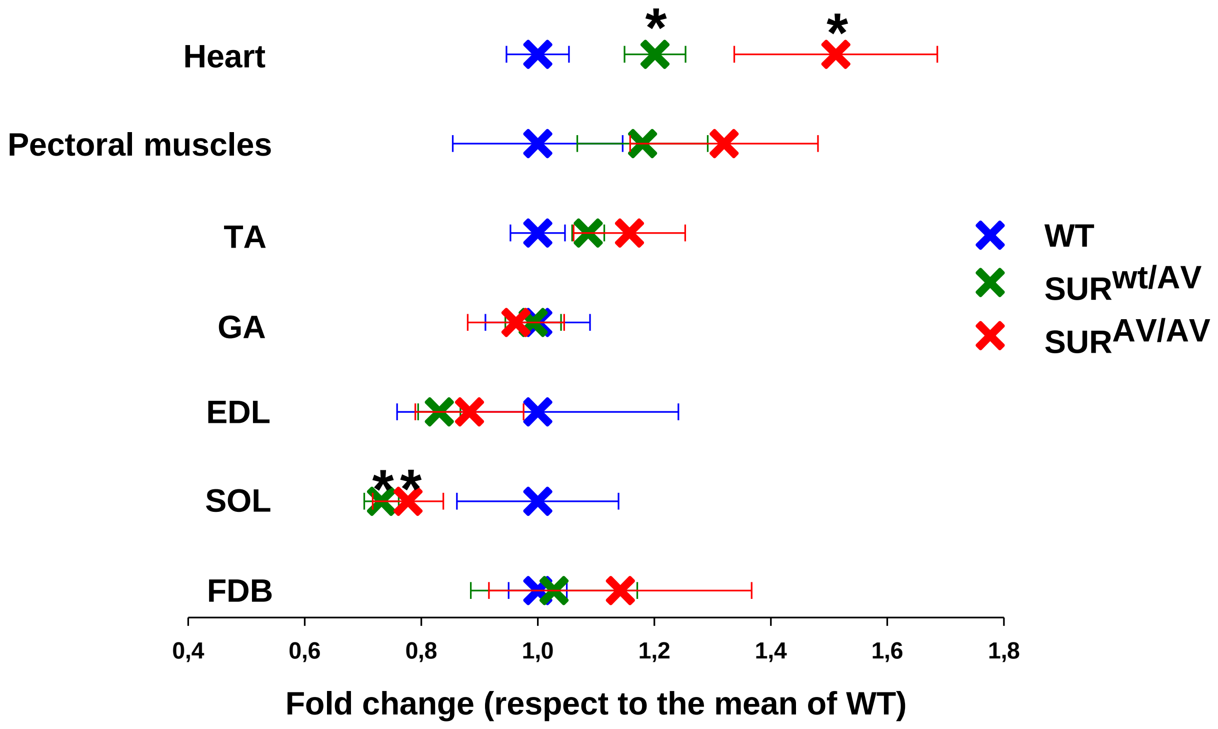

Figure S1. Organ weight differences between WT, SUR2$^{wt/AV}$ and SUR2$^{AV/AV}$ mice. Increased organ weight is observed in heart, pectoral muscles, Tibialis anterior (TA), and Flexor digitorum brevis (FDB) of SUR2$^{wt/AV}$ and SUR2$^{AV/AV}$ mice respect to the control. Soleus (SOL) was found less heavy in these SUR2 mutated mice. Values are presented as mean ± SEM (n. animal used for these evaluations: 4 animals per genotype); organ mean weights are normalized by the control mean. No differences were found in terms of weight among right and left muscles. GA, Gastrocnemius; EDL, Extensor digitorum longus. Data significantly different as evaluated with ONE WAY ANOVA and Bonferroni test (*p<0.05).
